# Inhibition of *Irvingia gabonensis *seed extract (OB131) on adipogenesis as mediated via down regulation of the PPARgamma and Leptin genes and up-regulation of the adiponectin gene

**DOI:** 10.1186/1476-511X-7-44

**Published:** 2008-11-13

**Authors:** Julius E Oben, Judith L Ngondi, Kenneth Blum

**Affiliations:** 1 Laboratory of Nutrition and Nutritional Biochemistry, Department of Biochemistry, B.P. 812, Faculty of Science, University of Yaoundé I, Yaoundé, Cameroon; 2 Department of Physiology & Pharmacology, Wake Forest University School of Medicine, Winston-Salem, North Carolina, USA

## Abstract

**Background:**

Endeavors to manage obesity have been heavily reliant on controlling energy intake and expenditure equilibrium, but have failed to curtail the overweight and obesity epidemic. This dynamic equilibrium is more complex than originally postulated and is influenced by lifestyle, calorie and nutrient intake, reward cravings and satiation, energy metabolism, stress response capabilities, immune metabolism and genetics. Fat metabolism is an important indicator of how efficiently and to what extent these factors are competently integrating. We investigated whether an Irvingia gabonensis seed extract (IGOB131) would provide a more beneficial comprehensive approach influencing multiple mechanisms and specifically PPAR gamma, leptin and adiponectin gene expressions, important in anti-obesity strategies.

**Methods:**

Using murine 3T3-L1 adipocytes as a model for adipose cell biology research, the effects of IGOB131 were investigated on PPAR gamma, adiponectin, and leptin. These adipocytes were harvested 8 days after the initiation of differentiation and treated with 0 to 250 microM of IGOB131 for 12 and 24 h at 37 degree C in a humidified 5 percent CO2 incubator. The relative expression of PPAR gamma, adiponectin, and leptin in 3T3-L1 adipocytes was quantified densitometrically using the software LabWorks 4.5, and calculated according to the reference bands of beta-actin.

**Results:**

The IGOB131 significantly inhibited adipogenesis in adipocytes. The effect appears to be mediated through the down-regulated expression of adipogenic transcription factors (PPAR gamma) [P less than 0.05] and adipocyte-specific proteins (leptin) [P less than 0.05], and by up-regulated expression of adiponectin [P less than 0.05].

**Conclusion:**

IGOB131 may play an important multifaceted role in the control of adipogenesis and have further implications in in-vivo anti obesity effects by targeting the PPAR gamma gene, a known contributory factor to obesity in humans.

## Background

Endeavors to manage obesity have been heavily reliant on controlling energy intake and expenditure equilibrium, but have failed to curtail the overweight and obesity epidemic. This dynamic equilibrium is more complex than originally postulated and is influenced by lifestyle, calorie and nutrient intake, reward cravings and satiation, energy metabolism, stress response capabilities, immune metabolism and genetics. Fat metabolism is an important indicator of how efficiently and to what extent these factors are competently integrating. Obesity is a condition in which adipocytes accumulate a large amount of fat and become enlarged. It is characterized at the cellular level by an increase in the number and size of adipocytes differentiated from fibroblastic preadipocytes in adipose tissue [1].

The adipocyte is the primary site for energy storage, which accumulates triglycerides due to factors that include nutritional excess (energy imbalance), nutrient deficiencies, excessive stress, and genetic predispositions among other causes. Shimomura et al. [2] indicated that adipocytes synthesize and secrete biologically active molecules called adipocytokines. During adipocyte differentiation, transcriptional factors such as peroxisome proliferator-activated receptor gamma (PPARγ) are involved in the sequential expression of adipocyte-specific proteins [3]. Adiponectin is an adipocytokine that has been shown to have antiatherogenic, anti-inflammatory, and antidiabetic roles [4]. It has been found to be an important modulator of insulin sensitivity [5]. Nakamura et al. [6] indicated that high circulating levels of adiponectin might be protective against the development of coronary artery disease. Adiponectin levels are inversely correlated to body fat percentage, indicating that adiponectin plays an important role in fatty acid catabolism. Yamauchi et al. [7] indicated that adiponectin has emerged most recently as an important adipocytokine with insulin-sensitizing effects and represents a novel treatment target for insulin resistance and type 2 diabetes. Leptin is a secreted protein hormone that affects the hypothalamus to inhibit food intake and stimulates thermogenesis [8]. The cytosolic enzyme Glycerol-3-Phosphate Dehydrogenase (G3PDH) appears to have an important role catalyzing the conversion of glycerol into triglyceride [9].

In the present study, we investigated the effects of a proprietary extract of OB131 *Irvingia gabonensis *(IGOB131) on the inhibition of intracellular triglyceride and G3PDH activity in 3T3-L1 adipocytes. We also examined the effect of these compounds on protein expression of adipogenesis in 3T3-L1 adipocytes.

## Methods

### Cell Culture

A murine 3T3-L1 cell line was used in this study due to its widespread acceptance as a cell model for adipose cell biology research over the course of several decades [10]. 3T3-L1 preadipocytes (BCRC 60159) were purchased from the Bioresource Collection and Research Center (BCRC, Food Industry Research and Development Institute, Hsinchu, Taiwan, ROC). 3T3-L1 preadipocytes were planted into 6-well plates and maintained in DMEM supplemented with 10% bovine calf serum at 37°C in a humidified 5% CO_2 _incubator. Adipocytic differentiation was induced by the adipogenic agents (0.5 mM IBMX, 1 μM DEX, and 1 μM INS) that were added to culture medium. Afterwards, the medium was changed to normal culture medium and was freshly replaced every 48 h. The cells were harvested 8 days after the initiation of differentiation.

### Triglyceride Content

Cells were incubated with 250 μM of IGOB131 for 72 h at 37°C in a humidified 5% CO_2 _incubator. Cells were collected and lysed in lysis buffer (1% Triton X-100 in PBS). The total triglyceride content in cells was determined using a commercial triglyceride assay kit (DiaSys Diagnostic Systems GmbH, Holzheim, Germany). The protein concentration was determined by using a BioRad DC protein assay kit (Bio-Rad Laboratories, Hercules, CA). Inhibition (%) was expressed as percent decrease in triglyceride content against control (0%).

### Glycerol-3-Phosphate Dehydrogenase Activity

3T3-L1 adipocytes were harvested 8 days after the initiation of differentiation and were incubated with 250 μM of IGOB131 for 72 h at 37°C in a humidified 5% CO_2 _incubator. Cells were washed twice with ice-cold PBS on 3T3-L1 adipocytes, and lysed in 25 mM Tris/1 mM EDTA, pH 7.5 for the measurement of glycerol-3-phosphate dehydrogenase (G3PDH) specific activity. G3PDH activity was determined according to the procedure of Wise and Green [11]. Protein concentration was determined by the BioRad DC protein assay kit (Bio-Rad Laboratories, Hercules, CA) using bovine serum albumin as a standard. Enzyme activity was expressed as units of activity/mg protein. Inhibition (%) was expressed as percent decrease in G3PDH activity against control (0%).

### Western Blot Assay

Cells were incubated with 0–250 μM of IGOB131 acids for 12 and 24 h at 37°C in a humidified 5% CO_2 _incubator. They were collected and lysed in ice-cold lysis buffer (20 mM tris-HCl (pH 7.4), 2 mM EDTA, 500 μM sodium orthovanadate, 1% Triton X-100, 0.1% SDS, 10 mM NaF, 10 μg/mL leupeptin and 1 mM PMSF). The protein concentration was estimated with the Bio-Rad DC protein assay (Bio-Rad Laboratories, Hercules, CA) using bovine serum albumin as a standard. Total protein (50–60 μg) was separated by sodium dodecyl sulfate-polyacrylamide gel electrophoresis (SDS-PAGE) using a 12% polyacrylamide gel. The proteins in the gel were transferred to a PVDF membrane. The membrane was blocked with 5% skim milk in PBST (0.05% v/v Tween-20 in PBS, pH 7.2) for 1 h. Membranes were incubated with primary antibody at 4°C overnight and then with secondary antibody for 1 h. Membranes were washed in PBST for 10 min three times between each step. The signal was detected using the Amersham ECL system (Amersham-Pharmacia Biotech, Arlington Heights, IL). The relative expression of PPARγ, adiponectin, and leptin in 3T3-L1 adipocytes was quantified densitometrically using the software LabWorks 4.5, and calculated according to the reference bands of β-actin.

### Statistical analysis

Values are expressed as mean_S.E. For multiple comparisons, a one-way analysis of variance (ANOVA) was used. When ANOVA showed significant differences, *post-hoc *analysis was performed with the Newman-Keuls multiple range test using SPSS.

## Results

### Effect of IGOB131 on the inhibition of Intracellular Triglycerides and G3PDH activity in 3T3-l1 adipocytes

The effect of IGOB131 on percent intracellular triglyceride and G3PDH levels were evaluated as indicated in the method section and the results are presented in Table 1. The reported values are the means ± SD of three samples. For this study cellular harvesting and incubation was accomplished with IGOB131 as previously described in the method section. We found that IGOB131 resulted in a significant inhibition of intracellular triglycerides (p < 0.05). The peak inhibition using 250 μM of IGOB131 for 72 h at 37°C in 5% CO_2 _incubator was 80.9 ± 0.7. Moreover we found a similar finding utilizing the same parameters for the intracellular G3PHD levels. We found that IGOB131 resulted in a significant inhibition of intracellular G3PDH (p < 0.05). The peak inhibition using 250 μM of IGOB131 for 72 h at 37°C in 5% CO_2 _incubator was 71.6 ± 1.2 (see Table 1).

**Table 1:** Effect of IGOB131 on the inhibition of Intracellular Triglycerides and G3PDH activity in 3T3-l1 adipocytes.

**Compound**	% Intracellular Triglyceride Inhibition*	**% Intracellular G3PDH Inhibition**
	
IGOB131	80.9 ± 0.7	71.6 ± 1.2

*Inhibitions (%) are expressed as percentages of the inhibition of control at 0%. 3T3-L1 adipocytes were harvested 8 days after the initiation of differentiation. The cells were incubated with 250 μM of IGOB131 for 72 h at 37°C in 5% CO_2 _incubator. The reported values are the means ± SD (*n *= 3).

### Effect of IGOB131 on protein levels of PPARγ, adiponectin, and leptin in 3T3-L1 adipocytes

#### PPARγ

**Figure 1:**
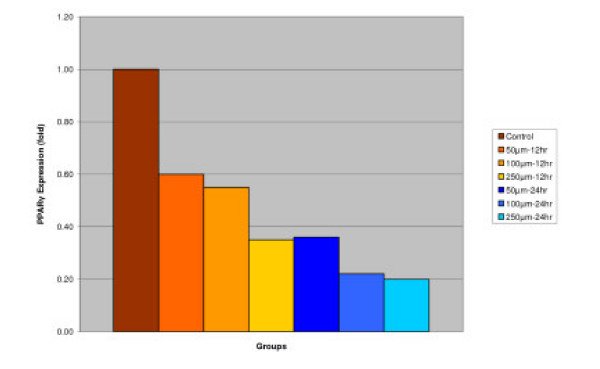
Effect of IGOB131 on protein levels of PPARγ in 3T3-L1 adipocytes. 3T3-L1 adipocytes were harvested 8 days after the initiation of differentiation. Cells were treated with 0–250 μM of IGOB131 for 12 and 24 h at 37°C in a humidified 5% CO_2 _incubator. The relative expression of PPARγ, in 3T3-L1 adipocytes was quantified densitometrically using the software LabWorks 4.5, and calculated according to the reference bands of β-actin. Values are means for three replicated cultures and *p < 0.05 vs. control.

Effect of IGOB131 on protein levels of PPARγ, adiponectin, and leptin in 3T3-L1 adipocytes. 3T3-L1 adipocytes were harvested 8 days after the initiation of differentiation. Cells were treated with 0–250 μM of IGOB131 for 12 and 24 h at 37°C in a humidified 5% CO_2 _incubator. The present experiment indicated that IGOB131 treatment significantly (P < 0.05) inhibited the expression of PPARγ protein levels (Figure 1).

#### Leptin

##### Effect of IGOB131 on protein levels of leptin in 3T3-L1 adipocytes

**Figure 2:**
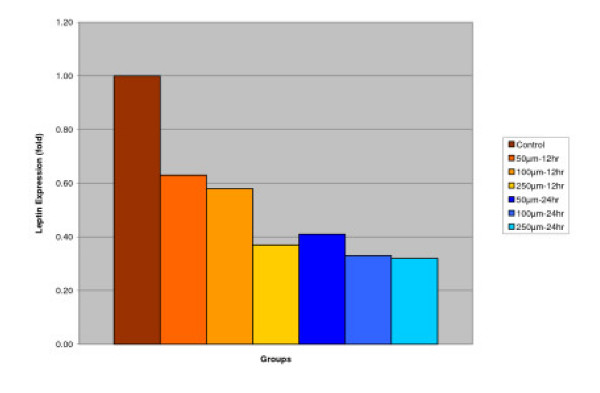
Effect of IGOB131 on protein levels of Leptin in 3T3-L1 adipocytes. 3T3-L1 adipocytes were harvested 8 days after the initiation of differentiation. Cells were treated with 0–250 μM of IGOB131 for 12 and 24 h at 37°C in a humidified 5% CO_2 _incubator. The relative expression of Leptin, in 3T3-L1 adipocytes was quantified densitometrically using the software LabWorks 4.5, and calculated according to the reference bands of β-actin. Values are means for three replicated cultures and *p < 0.05 vs. control.

3T3-L1 adipocytes were harvested 8 days after the initiation of differentiation. Cells were treated with 0–250 μM of IGOB131 for 12 and 24 h at 37°C in a humidified 5% CO_2 _incubator. In the present study, IGOB131 reduced the demand for excessive leptin synthesis, reducing circulating serum leptin levels (P < 0.05). (Figure 2)

#### Adiponectin

##### Effect of IGOB131 on protein levels of Adiponectin in 3T3-L1 adipocytes

**Figure 3:**
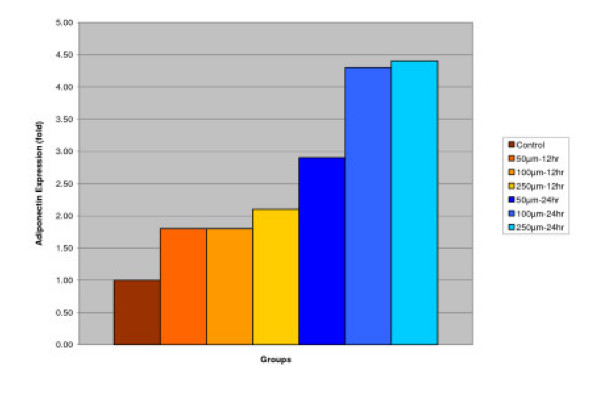
Effect of IGOB131 on protein levels of Adiponectin in 3T3-L1 adipocytes. 3T3-L1 adipocytes were harvested 8 days after the initiation of differentiation. Cells were treated with 0–250 μM of IGOB131 for 12 and 24 h at 37°C in a humidified 5% CO_2 _incubator. The relative expression of Adiponectin in 3T3-L1 adipocytes was quantified densitometrically using the software LabWorks 4.5 and calculated according to the reference bands of β-actin. Values are means for three replicated cultures and *p < 0.05 vs. control.

3T3-L1 adipocytes were harvested 8 days after the initiation of differentiation. Cells were treated with 0–250 μM of IGOB131 for 12 and 24 h at 37°C in a humidified 5% CO_2 _incubator. In the present study, IGOB131 up-regulated the expression of Adiponectin (P < 0.05). (Figure 3)

## Discussion

Over the past few decades, obesity has become a global epidemic in both developed and developing countries. It is characterized by an increased adipose tissue mass and is associated with high health risk [1]. The prevalence of obesity and obesity-related disorders has led to major research interests in the influence of adipose tissue mass [12]. The 3T3-L1 cell line is widely used as a model of adipocyte differentiation and adipose biology. Wang and Jones [13] indicated that the decreased adipocytic lipogenesis is one of the mechanisms of proposed antiobesity. In the present study, we focused on the effects of IGOB131 on inhibiting adipogenesis in 3T3-L1 adipocytes. The inhibitory effect resulted from the repression of adipocyte-specific protein expressions.

The goal of this research was to study the inhibition of adipogenesis and adipocyte differentiation with IGOB131. We investigated the effects of IGOB131 on the inhibition of intracellular triglyceride and G3PDH activity in 3T3-L1 adipocytes. Fasting induces conversion of glycerol into triglyceride through an induction of several hepatic enzymes such as G3PDH and glycerol kinase. Tomiyama et al. [14] indicated that the expression of G3PDH is induced several fold upon conversion of preadipocytes to adipocytes, which is the predominant substrate for triglyceride synthesis in adipose tissue. Our data indicated that the exposure of 3T3-L1 adipocytes to IGOB131 resulted in lower levels of intracellular triglycerides and G3PDH than other compounds tested (Table 1).

Adipose tissue is now known to produce and secrete a PPARγ, which has roles in the early stage of adipocyte differentiation, because they are transcriptional factors for numerous genes [14,15]. Some studies have addressed the important role that PPARγ plays in the regulation of insulin sensitivity and glucose homeostasis [16]. The present experiment indicated that IGOB131 treatment inhibited the expression of PPARγ protein levels (Figure 1), which demonstrated that adipogenesis was inhibited by affecting the transcriptional factor cascade upstream of PPARγ expression. Leptin (product of ob gene) that is secreted from adipocytes and gains access to the brain, reduces food intake, and increases energy expenditure [7]. Leptin that is unable to gain access to the brain, due to CRP binding resulting in leptin resistance, increases hypothalamic signaling for leptin synthesis, promoting higher levels of circulating serum leptin. Adiponectin is specifically expressed in white adipose tissues and is one of the most important adipocytokines. Adiponectin is an adipocytokine that has been shown to have antiatherogenic, anti-inflammatory and antidiabetic roles [5]. In the present study, IGOB131 reduced the demand for excessive leptin synthesis, reducing circulating serum leptin levels, and stimulated the up-regulation of adiponectin at the protein level (Figures 2, 3). Adiponectin expression would, therefore, be regulated by PPARγ transcriptional activity [17].

## Conclusion

The inhibitory effects of IGOB131 on 3T3-L1 adipocytes, as indicated by the decrease in intracellular triglyceride content and G3PDH activity have been elucidated. It appears to be mediated through the down-regulated expression of adipogenic transcription factors (PPARγ) and adipocyte-specific proteins (leptin), and then the up-regulated expression of adiponectin. These results indicate that IGOB131 may play an important role in the control of adipogenesis and might have further implication in *in-vivo *antiobesity effects that exert specific influence on the PPARγ gene, a known contributory factor to obesity in humans [18]. This research provides insight into an important mechanism for combating obesity.

## Authors' contributions

Julius Oben supervised the study, Judith Ngondi carried out some analyses, Kenneth Blum and Julius Oben synthesized the data, interpreted the results and wrote the manuscript.
